# Preparation and Characterization of High Surface Area Activated Carbon Fibers from Lignin

**DOI:** 10.3390/polym8100369

**Published:** 2016-10-18

**Authors:** Jian Lin, Guangjie Zhao

**Affiliations:** 1 Beijing Key Laboratory of Wood Science and Engineering, Beijing Forestry University, Beijing 100083, China; linjian0702@bjfu.edu.cn; 2 Zhejiang Province Key Laboratory of Wood Science and Technology, Zhejiang University of Agriculture and Forestry, Hangzhou 311300, China

**Keywords:** lignin, activated carbon fibers, porous structure, methylene blue

## Abstract

Activated carbon fibers (ACFs) were successfully prepared from softwood lignin, which was isolated with polyethylene glycol 400 (PEG-400) as a solvolysis reagent, by water steam activation. The pore characterization and adsorption property of ACFs were investigated. The results showed that all the ACFs with more micropores exhibited high specific surface area and total pore volume which increased with the activation time prolonging; the highest ones were around 3100 m^2^/g and 1.5 mL/g, respectively. The specific surface area and total pore volume were much larger than those of other types of lignin-based ACFs and activated charcoal. Besides, with increasing activation time, the amount of graphitic carbon, which was the main compound on the surface of ACFs, decreased, while the amount of functional groups containing C–O slightly increased. In addition, the adsorption capacity of ACFs for methylene blue was highly increased as the activation time increased. Accordingly, lignin isolated with PEG is a promising precursor for ACF production.

## 1. Introduction

Activated carbon fibers (ACFs) with large amounts of well-defined porous structures were usually prepared from carbon fibers by activation with some gas or chemical reagent. They could be widely utilized in several fields, such as purification, separation, electronic materials, catalysts and storage of natural gas [1,2], due to their large specific surface area and pore volume. In general, pitch [3], polyacrylonitrile [4], viscose rayon [5], and phenolic resin [6] are used as raw materials to commercially manufacture the ACFs by the procedures of spinning, thermostabilization, carbonization, and activation. However, such petrochemical precursors are costly and not environmentally friendly, because of the decreasing reserves of fossil fuels and the resultant negative effects of their use. Thus, more and more attention was paid to the biomass resources as low-cost and renewable raw materials for fabricating ACFs due to their abundance and sustainability.

Lignin, one of the major cell wall components in higher plants, is the second most abundant renewable bio-resource next to cellulose on the earth. It was usually obtained as a by-product from pulp and paper-making processes. With the concept of the biorefinery proposed, high value-added utilization of lignin seems to be more significant. Considering the high carbon content, lignin has been applied to synthesize carbonaceous materials such as carbon fibers [7,8,9,10,11,12,13,14,15,16]. However, as a result of isotropic structure, lignin-based carbon fibers showed poor mechanical properties and had difficulty meeting the requirement of general grade use. Nevertheless, this structure favors forming pores in lignin-based carbon fibers during the activation process, resulting in the preparation of ACFs with high specific surface area and pore volume [17,18].

Furthermore, a solvolysis process using polyethylene glycol (PEG) was considered as a candidate for use in terms of biorefinery to obtain levulinic acid from wood [19]. As a by-product of the solvolysis, a lignin fraction could be obtained, and it was found to be chemically modified with PEG through the formation of ether bonds between the PEG moiety and lignin. This lignin exhibited good fusibility and was further processed into carbon fibers. However, the mechanical properties of the resultant carbon fibers were quiet low because of the existing defects including a porous and flabby structure.

The defect was disadvantage for preparing carbon fibers with excellent mechanical properties, but it could be used as an advantage to easily form more pores during the activation process. Accordingly, in this article we describe the efforts directed at the preparation and characterization of lignin-based activated carbon fibers by water steam activation with different activation times.

## 2. Materials and Methods

### 2.1. ACFs Preparation

Lignin powder was isolated from cedar wood chips. Briefly, 60 g of dried cedar wood chips were cooked with 300 g of polyethylene glycol 400 (PEG 400) and 0.9 g of 95% aqueous sulfuric acid (0.3 wt % on PEG 400) at 160 °C for 240 min. After cooling to room temperature with an ice water bath, 1.2 L of 80% 1,4-dioxane aqueous solution was added to the reaction mixture. The resultant mixture was filtrated through filter paper (No. 5C, Advantec, Shanghai, China) to separate filtrate and residue. The 1,4-dioxane in the filtrate was removed by evaporation under reduced pressure. The resulting residual liquor was boiled at 140 °C for 150 min. When finished, the solution was dropped into 4 L of distilled water with continuous stirring for 30 min. The precipitate was then collected by centrifugation at 11,600 G for 15 min. Finally, lignin powder was obtained by freeze drying.

Subsequently, lignin powder was spun into lignin-based fibers by using a laboratory spinning apparatus with a single hole nozzle (diameter: 0.8 mm) at the nozzle temperature of 172 °C and the winding rates of 67 m/min under a nitrogen pressure. Then, lignin-based fibers were immersed in aqueous HCl solution at concentrations of 6 M at 100 °C for 2 h. After the treatment, the resultant fibers were washed twice with distilled water, and then roughly dried in an oven at 105 °C for 2 h. When finished, the resultant lignin-based fibers were carbonized in the electronic muffle furnace (KDF S90/S90G, Denken Co. Ltd., Kyoto, Japan) from room temperature to 1000 °C at a heating rate of 3 °C/min and then held at 1000 °C for 1 h under a N_2_ stream at a flow rate of 0.15 L/min to yield lignin-based carbon fibers (CFs).

The resultant CFs was subjected to activation to prepare ACFs. The CFs were heated in the same electronic muffle furnace (used in carbonization step) from room temperature to 900 °C at a heating rate of 10 °C/min under a N_2_ stream (flow rate, 0.6 L/min). Subsequently, N_2_ was mixed with steam (60.3 RH%) and the mixed gas was introduced for activation at the final temperature of 900 °C for 30 to 90 min.

### 2.2. Characterization

#### 2.2.1. Scanning Electronic Microscopy (SEM)

The surface morphology of ACFs were observed using scanning electron microscopy (SEM, S-3400N, Hitachi Co. Ltd., Tokyo, Japan) at magnifications of 150 to 2000× and at an accelerating voltage of 5 kV. Before observation, the samples were coated with a thin layer by spraying gold metal using Ion Sputter (E-1010, Hitachi Co. Ltd., Tokyo, Japan) before searching morphology. 

#### 2.2.2. Fourier Transform Infrared Spectroscopy (FTIR)

The chemical groups of ACFs were examined using FTIR spectrum analysis with GX FT-IR system (PerkinElmer, Norwalk, CA, USA) in the scanning range of 4000 to 400 cm^−1^. The samples were pulverized using size 100 mesh and mixed with potassium bromide at the ratio of 1:100, before being pressed into a disk. 

#### 2.2.3. Porous Measurement

N_2_ adsorption-desorption isotherms were determined at 77 K by a surface area analyzer (Autosorb-iQ, Quantachrome Instruments Japan GK, Kanagawa, Japan). Prior to measurement, the samples were preheated at 300 °C for 3 h under vacuum to remove any moisture or volatiles within the existing pores of the materials. The Brunauer, Emmett, and Teller (BET) model was used to calculate the surface area (*S*_BET_) of the samples from N_2_ adsorption isotherms in the range of the relative pressure (*P*/*P*_0_) from 0.05 to 0.30 [20]. The adsorption total pore volumes at relative pressures of 0.1 and 0.995 are equal to the volume of micropores, and the total volume (*V*_tot_) of micropores and mesopores, respectively [21,22,23]. The micropore area (*S*_micro_) and mesopore area (*S*_meso_) were calculated according to the t-plot method and Barrett–Joyner–Halenda (BJH) method, respectively. The pore size distribution (PSD) was calculated using the density functional theory (DFT) method [24], which is based on the calculated N_2_ adsorption isotherms for different pore sizes.

#### 2.2.4. X-ray Photoelectron Spectroscopy (XPS)

X-ray photoelectron spectroscopy measurements were carried out on a spectrophotometer (ESCALAB 250Xi, Thermo Fisher Scientific Inc., Waltham, MA, USA) to determine the number and type of functional groups present on the surface of ACFs with a monochromated Al Ka X-ray source (*hν* = 1486.6 eV). A current of 10 mA and a voltage of 13 kV were used. The survey scans were collected from the binding energy range of 0 to 1350 eV. A nonlinear, least squares regression analysis program (XPSPEAK software, Version 4.1., Informer Technologies, Inc., Hong Kong, China) was used for the XPS spectral deconvolution.

#### 2.2.5. Methylene Blue Adsorption Capacity

Liquid-phase adsorption tests for ACFs were conducted, using methylene blue (MB, CAS 7220-79-3, Tianjin Jinke Fine Chemicals CO. Ltd., Tianjin, China) as the adsorbate. Firstly, methylene blue solution with four different concentrations (0.24, 0.48, 1.2, and 2.4 mg/L) were prepared, and the adsorption values of each methylene blue solution were taken at 630 nm from UV spectrometer (UV-1800, Shimadzu, Kyoto, Japan). These results were measured for drawing the standard calibration curve.

Subsequently, samples were mixed with 25 mL of methylene blue solution (1200 mg/L) in a 100 mL of conical flask. The mixture was shaken for 30 min in a water bath (20–30 °C) with a shaking rate of 150 rpm. After shaking, the mixture was filtered and the filtrate was scanned by UV spectrometer. The adsorption values at 665 nm were compared with the standard calibration curve to obtain the final concentration of methylene blue after adsorption. The adsorption capacity was estimated by the following formula:

*Q* = [(1200 − *C*) × 25/1000]/*S* (1)

where: *Q*—Adsorption capacity, mg/g; *C*—Final concentration of methylene blue after adsorption, mg/L; *S*—Sample weight, g.

Finally, at least two replications were carried out for each sample to obtain the different methylene blue solution concentrations which were higher and lower 0.24 mg/L, respectively. Calibration line of the adsorption capacity as a function of the methylene blue concentration was obtained to calculate the methylene blue adsorption capacity of the corresponding sample when at the concentration of 0.24 mg/L [25,26].

## 3. Results

### 3.1. Yield and Morphologies of ACFs

Lignin-based ACFs were prepared from lignin powder as a raw material by melt-spinning and acid treatment and carbonization as well as water steam activation at the temperature of 900 °C with different activation times. Generally, an efficient ACF production process combines an acceptable fabrication yield. In this study, the yield of PEG-lignin was 50.4% on wood chips. However, its PEG content was 43.8%. Accordingly, the net lignin based on wood was estimated to be 28.3%. Furthermore, the ACF production yields were monitored for the three used activating times. The yield percentages of ACFs are shown in Table 1. The yield decreased from 54% to 13% with the increasing activation time. This tendency was consistent with the results from the preparation of activated carbon fibers from acetic acid lignin [17,18]. The decrease in yield may be caused by the reaction of steam with carbon, and the longer steam activation time resulted in a higher burn off as well as more pores. Therefore, more porous structures in ACFs could be observed from the SEM morphologies.

**Figure 1:**
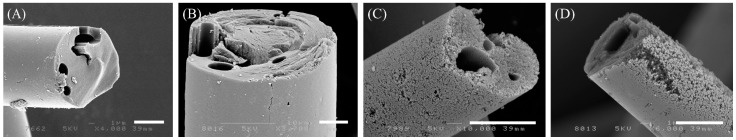
SEM morphologies of CFs (**A**) and ACFs obtained from CFs activated at 900 °C for (**B**) 30 min; (**C**) 60 min; (**D**) 90 min (white bar = 5 μm).

Figure 1 shows the SEM morphologies of the resultant ACFs. As shown in Figure 1A, CFs without activation presented the split and defects on the surface, which were caused by the removal of the PEG moiety from lignin fibers during chemical thermostabilization. After steam activation, the porous and flabby structure still existed on the surface of ACFs. With an increase in the activation time, more pores seemed to be developed on the surface and cross-section of the ACFs. Especially for the activation times of 60 and 90 min, the morphologies were obviously changed. A much rough surface and pores can be easily and clearly observed. These results were also quantitatively proved by nitrogen adsorption-desorption measurements.

### 3.2. Pore Structure of ACFs

**Figure 2:**
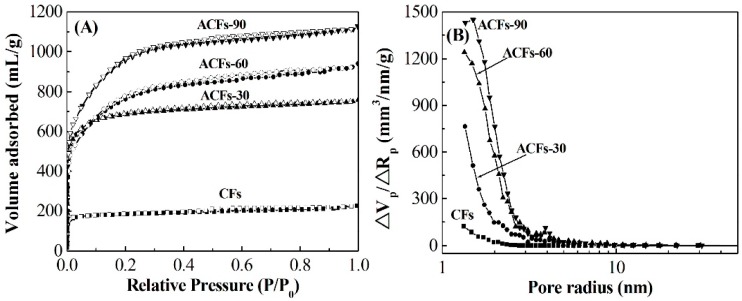
N_2_ adsorption-desorption isotherms at 77 K (**A**) and pore size distribution; (**B**) of CFs and ACFs prepared with various activation times, respectively.

The isotherms of N_2_ adsorption-desorption at 77 K of CFs and of the ACFs obtained are shown in Figure 2A. Compared with all the ACFs, CFs exhibited a small volume of around 200 mL/g of adsorbed N_2_. The major uptake of N_2_ occurred at a low relative pressure (*P*/*P*_0_ < 0.05) and then reached the plateau at a high relative pressure, implying that CFs may have more micropores but fewer mesopores. After activation treatment, ACFs-30 showed the same isotherm as type I according to the IUPAC (International Union of Pure and Applied Chemistry) classification [27], but a larger volume of adsorbed N_2_ with CFs. More than 600 mL/g of N_2_ was adsorbed by ACFs-30, which was over three times larger than that of CFs. This means that more micropores were developed in ACFs-30 in comparison with CFs. With the activation time increasing, ACFs-60 and ACFs-90 presented a greater volume of adsorbed N_2_ and different isotherms, which had a sharp increase and then a glacis knee followed by the slope of the plateau with hysteresis loops at the relative pressure of 0.4–0.9 due to the multilayer adsorption phenomenon on the surface of the adsorbent and possibly capillary condensation in the mesopores, suggesting the coexistence of a larger amount of micropores and mesopores.

Details on the pore size distribution of the ACFs produced are presented in Figure 2B. Obviously, almost no mesopores could be found and micropores were mainly distributed in CFs. With the increase of the activation time, the porous developments of the ACFs obtained were extremely uniform. More micropores with a small amount of mesopores were generated, suggesting that the water steam activation not only produced new micropores but also widened them even to mesopores by removing the active amorphous atoms and unsaturated carbon atoms from the edges of the micro-graphitic walls and partially gasifying the micropores’ walls [28,29]. This phenomenon also can be interpreted by the average pore diameter changes of ACFs (Table 1), which were ranging from 1.9 to 2.4 nm. Besides, the average pore diameters were comparable to that of hardwood acetic acid lignin–based ACFs, and smaller than that of softwood acetic acid lignin–based ACFs. This may result in the high specific surface area.

As shown in Table 1, the *S*_BET_ and *V*_tot_ of ACFs obtained from the N_2_ adsorption isotherms were gradually increased with the activation time increasing. The corresponding specific surface areas of ACFs were 2064, 2490, and 3110 m^2^/g, respectively, which were much larger than those of ACFs prepared from hardwood acetic acid lignin (1250 m^2^/g) [17] and softwood acetic acid lignin (1930 m^2^/g) [18]. This result indicated that the defects on the surface of CFs contributed to the formation of the porous structure. The specific surface areas of ACFs were also larger than or equal to those of other types of ACFs prepared from rayon (1635 m^2^/g) [30], PAN (1320 m^2^/g) [31], pitch (2400 m^2^/g) [32] and phenolic resin (~2500 m^2^/g) [33], suggesting that the lignin isolated from wood chips with PEG is a promising precursor for the preparation of ACFs. 

### 3.3. Chemical Compositions of ACFs

**Figure 3:**
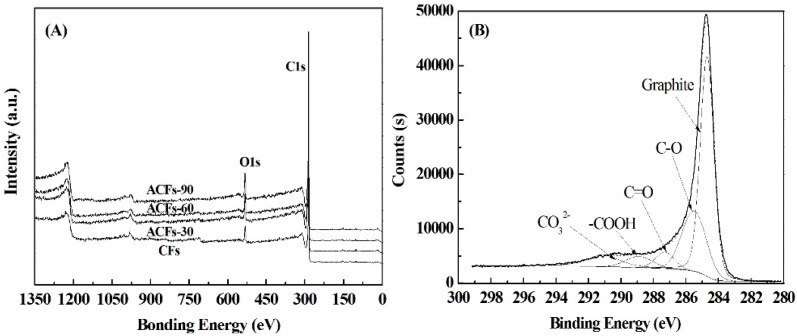
X-ray photoelectron spectroscopy spectra of CFs and ACFs (**A**); and of C1s region for ACFs-90 (**B**).

The XPS spectra of CFs and prepared ACFs are shown in Figure 3A. The major peaks in the spectra are attributed to C1s and O1s photoelectrons. Table 2 summarizes the elemental compositions on the surface of the samples. The contents of N and S atoms were quite low, and both of the samples were smaller than 1 at. % (atomic percent). Especially for elemental S, only 0.1 at. % was found. Elemental C was the most abundant constituent in all of the samples, and the carbon content slightly increased with the increase in activation time, except for ACFs-90. The longer the activation time, the more violent the reaction between carbon and steam took place in the ACFs, resulting in the oxidation of elemental C and then decreasing the carbon content. That is also why the oxygen content increased after the activation time was increased to 60 min.

The C1s spectra for ACFs were very similar. Therefore, only the spectrum for ACFs-90 is presented in Figure 3B, as an example. For all of the ACFs, the C1s signals exhibited an asymmetric tailing, which was partially attributed to the intrinsic asymmetry of the graphite peaks or to the contribution of oxygen surface complexes. Deconvolution of the XPS C1s spectra produced five individual component peaks, representing graphitic carbon (284.6 to 284.7 eV); carbon present in alcohol, ether, or C=N groups (285.5 to 286.2 eV); carbonyl or quinine groups (286.9 to 287.1 eV); carboxyl, lactone, or ester groups (288.1 to 288.9 eV); and/or carbonate groups (290.7 eV) [34].

Table 3 summarizes the percentages of graphitic and functional carbon atoms. The values in % intensity for graphitic carbon and oxygen-containing groups showed obvious differences among ACFs. After activation, the relative amount of the graphitic carbon in ACFs decreased as the activation time increased, whereas the C/O functional groups, except for carbonyl, changed in the opposite direction. In addition, the percentage of carbonate groups increased notably with the increasing activation time. It is believed that more graphitic carbons of ACFs reacted with steam molecules to generate more functional groups containing C–O.

**Figure 4:**
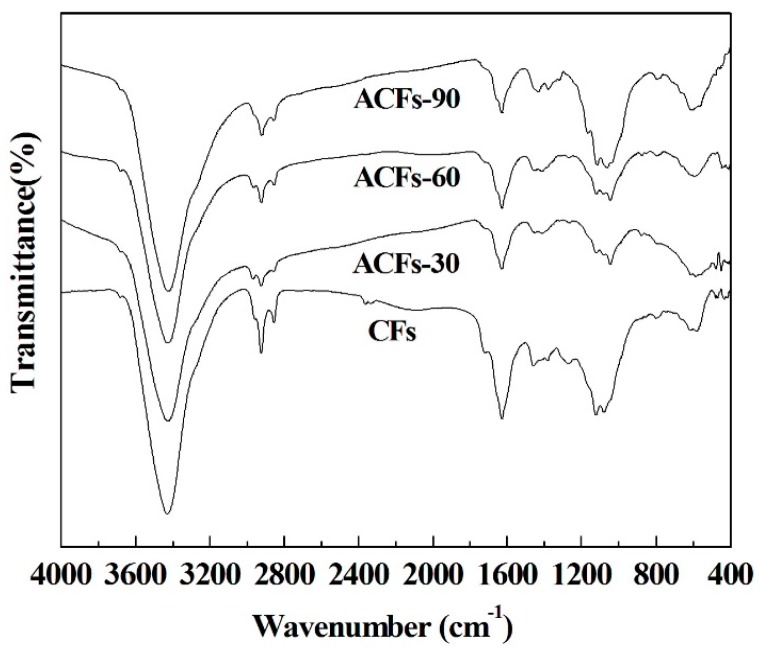
Fourier transform infrared spectroscopy of CFs and of ACFs prepared by different activation time.

Furthermore, Figure 4 illustrates the FTIR spectra of CFs and ACFs prepared at various activation times. The FTIR spectrum of CFs reveals an adsorption peak at 3431 cm^−1^, attributed to the –OH vibration frequencies of hydroxyl groups [35]. After activation, slightly higher intensities and broader signals were obtained, indicating a small increase in the number of –OH groups present in the ACFs. Bands for the C–H stretching of methylene groups (2923 cm^−1^) almost disappeared in the FTIR spectra of ACFs. Bands for conjugated C=O stretching vibrations of 1628 cm^−1^ showed decreased intensities with the increasing activation time [36]. Moreover, the intensity of the adsorption peaks near 1119 cm^−1^, assigned to the C–O stretching vibration, increased, indicating the abundance of oxygen-containing functional groups within the ACFs. Thus, the FTIR spectra of all ACFs showed fewer intense absorption bands than those of the CFs, revealing that the activation process with steam and heat facilitated the restructuring of the carbon structure by the formation or vaporization of volatile materials [37]. These results are consistent with those of XPS. 

### 3.4. Adsorption of Methylene Blue

**Figure 5:**
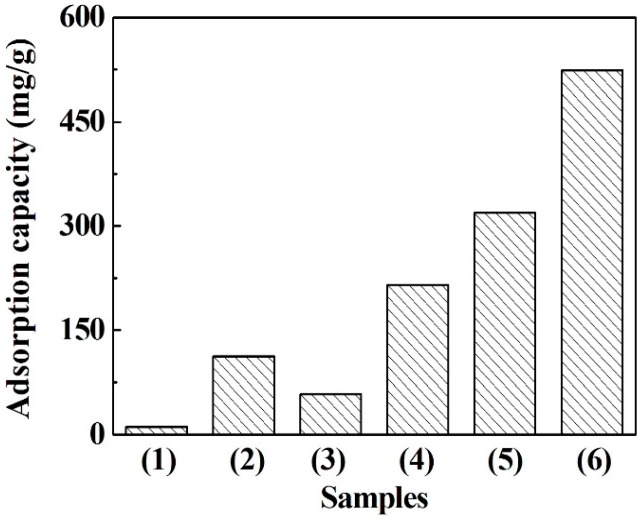
Methylene blue adsorption capacity of various carbon materials. (**1**) Charcoal; (**2**) Activated charcoal; (**3**) CFs; (**4**), (**5**) and (**6**) ACFs treated with 30, 60 and 90 min activation, respectively.

The liquid-phase adsorption tests for CFs and ACFs were conducted using methylene blue as the adsorbate. Figure 5 shows the adsorption capacities of various carbon materials against methylene blue. Evidently, ACFs showed a higher adsorption capacity for methylene blue than that of the commercial charcoal and activated charcoal. With the rise of the activation time, the adsorption capacity of ACFs gradually increased. Especially for the ACFs derived from 90 min activation, the highest adsorption capacity of 524 mg/g was obtained, which was around nine times greater than that of CFs. This means the activation process obviously improved the adsorption capacity, which is consistent with the results of the specific surface area between CFs and ACFs. Besides, the methylene blue adsorption value of ACFs in this study was also much higher than that of acetic acid lignin–based ACFs [18] and other ACFs from various raw materials in previous studies [38]. Furthermore, the yields of ACFs in this study were also comparable to that of other ACFs from different raw materials. Consequently, PEG-lignin could be considered as a promising alternative for the preparation of ACFs with high adsorption capacity.

## 4. Conclusions

Lignin isolated from softwood chips with polyethylene glycol 400 (PEG-400) as a solvolysis reagent was successfully converted into ACFs with water steam activation. The defects including a porous and flabby structure in the carbon fibers accelerated the generation of more micropores and a small amount of mesopores during the activation process. The ACFs with the maximum surface area of around 3100 m^2^/g and a total pore volume of around 1.5 cm^3^/g were obtained in this work, respectively. Besides, with an increase of the activation time, the amount of graphitic carbon decreased while the amount of functional groups containing C–O slightly increased. Accordingly, the obtained ACFs showed high methylene blue adsorption values in this study, and could be used as a very promising adsorbent for pollution control.

## Figures and Tables

**Table 1:** Yield and nitrogen adsorption (properties of CFs carbon fibers) and ACFs (Activated carbon fibers).

Samples	Yield ^1^ (%)	*S*_BET_ (m^2^/g)	*S*_micro_ (m^2^/g)	*S*_meso_ (m^2^/g)	*V*_tot_ (mL/g)	*D* ^2^ (nm)
CFs	100	775	741	34	0.410	1.9
ACFs-30	54.4	2064	1910	127	1.029	2.3
ACFs-60	19.5	2490	2200	246	1.153	2.4
ACFs-90	12.9	3110	2830	231	1.472	2.3

^1^ The yield of ACFs was based on CFs; ^2^ The average pore diameter.

**Table 2:** Elemental composition of CFs and prepared ACFs determined by XPS (X-ray photoelectron spectroscopy).

Samples	C (%)	O (%)	N (%)	S (%)	O/C (%)
CFs	93.5	5.7	0.7	0.1	6.1
ACFs-30	93.9	5.6	0.5	0.1	6.0
ACFs-60	94.3	5.1	0.6	0.1	5.4
ACFs-90	93.5	6.0	0.5	0.1	6.4

**Table 3:** Results of the fits of the C1s regions ^1^.

Samples	Graphite C–C (CP1)	C–OH (CP2)	C=O (CP3)	C–OOH (CP4)	CO_3_^2−^ (CP5)
CFs	68.5	20.2	8.0	1.9	1.4
ACFs-30	58.7	21.2	8.1	3.6	8.3
ACFs-60	57.2	21.7	7.7	4.0	9.4
ACFs-90	55.7	25.2	5.4	5.2	8.4

^1^ Values given in % of total intensity.
